# Outcome of Treatment in Children With Chronic Viral Hepatitis C: A Single Centre Study

**DOI:** 10.7759/cureus.21073

**Published:** 2022-01-10

**Authors:** Amima Usman, Iqtadar Seerat, Sana Batool Rizvi, Sarah Sheraz, Hafiz Aamir Yousaf

**Affiliations:** 1 Pediatric Gastroenterology and Hepatology, Pakistan Kidney and Liver Institute and Research Centre, Lahore, PAK

**Keywords:** interferon-alpha, treatment, sustained viral response, hepatitis-c infection, genotype

## Abstract

Objective

To review the efficacy of the combination of pegylated interferon-α 2b and ribavirin, and sofosbuvir and ribavirin in achieving sustained viral response (SVR) in chronic hepatitis C genotypes 1 and 3 in children.

Methods

A retrospective descriptive study was performed for children under 15 years of age treated for chronic hepatitis C at Pakistan Kidney and Liver Institute and Research Centre, Lahore, between 2018 and 2019. Demographic data and clinical information were collected. In addition, treatment outcome was assessed by SVR, defined as the absence of detectable viral RNA in blood after 24 weeks of initiation of treatment.

Results

A total of 30 children aged 15 years and below were included in this study. Sixteen of 30 children were males, and 14 were females. Of these 30 patients, four had hepatitis C virus (HCV) genotype 1, and 26 children had HCV genotype 3. The children with genotype 1 (a+b) were given the combination of ribavirin and pegylated interferon alfa-2b (Peg-IFN-α-2b). The remaining with HCV genotype 3 were given the combination of ribavirin and sofosbuvir for 24 weeks. Overall, 27 out of 30 (90%) children attained SVR at six months (100% children with genotype 1 and 88.4% children with genotype 3).

Conclusion

The combined therapy of ribavirin and sofosbuvir or Peg-IFN-α-2b and ribavirin is highly effective in treating chronic HCV infection in children.

## Introduction

Hepatitis C virus (HCV) is a serious global health concern since it causes chronic liver disease and related complications if left untreated. Approximately 2.1 to 5 million children worldwide have been ill with HCV to date [1]. Prevalence in children ranges from 0.05% to 0.36% in the United States [2]. HCV is highly endemic in Pakistan. According to general analysis steered in 2007-2008, the HCV prevalence in children under 15 years is at 4.8% [1,3]. However, the data regarding response to HCV treatment in children and pediatric management guidelines are relatively scanty [4]. The combination of sofosbuvir with ledipasvir or ribavirin to treat HCV genotype 3 is approved by the European Medicines Agency (EMA) and the FDA. The current guidelines recommend these agents in youths aged 12 years and above [5]. In comparison, the combination of interferon-alpha 2b and ribavirin has been given more effectively in genotype 1 or to children below 12 years of age. The aim of this study was to assess the efficacy and safety of pegylated interferon alfa-2b (Peg-IFN-α-2b) plus ribavirin and sofosbuvir-ribavirin in children with chronic hepatitis C.

This article was previously presented as an E-poster in 1st Virtual Research Moot at Pakistan Kidney and Liver Institute and Research Centre, Lahore, Pakistan on January 1, 2021, and out of the total of 51 abstracts submitted, it got the first prize.

## Materials and methods

Children aged ≤15 years and living in Punjab were initially screened for viral hepatitis at Pakistan Kidney and Liver Institute and Research Centre, Lahore, Pakistan, from March 2018 to March 2019. A retrospective descriptive study was carried out at the Paediatric Gastroenterology and Hepatology Department of Pakistan Kidney and Liver Institute and Research Centre.

A sample size of 30 children was taken, who were ≤15 years of age, whose HCV treatment was completed using the non-probability purposive sampling technique. Children over the age of 15 were excluded from the study. Ethical approval was taken by the Institute Research Board (IRB). Consent was obtained from the parents of the included children to obtain data and they were explained the purpose of this research study. Data were collected from PKLI software and questionnaire proformas and analyzed by SPSS version 20. We calculated frequencies and percentages to see the outcome of the treatment given. All quantitative variables were presented in mean ± SD to see the pre and post effect in the two groups. We used a non-parametric Wilcoxon signed-rank test, and p-value <0.05 was considered statistically significant.

## Results

The appraised data of 30 children who fulfilled inclusion criteria showed that 16 of 30 children (53.33%) were males and 14 (46.67%) were females. A total of 27 patients belong to the age group 11-15 years and three aged 5-10 years. Of the subjects assessed, four patients (13.3%) also had co-infection with viral hepatitis B. Out of 30 children, 12 patients (40%) had a family history of viral hepatitis B or C. Of 30, mothers of two children were hepatitis B virus (HBV) positive, mothers of seven children were HCV positive. In addition, both parents of two children were HCV positive, and both parents of one child were HBV and HCV positive (Table 1).

**Table 1:** Demography. HBV: Hepatitis B virus.

	Age			Gender		Genotype		Co-infection with HBV		Family History	
	0-5	6-10	11-15	Male	Female	1	3	Yes	No	Yes	No
Number	0	3	27	16	14	4	26	4	26	12	18
Percentage	0	10	90	53.3	46.6	13.3	46.6	13.3	86.6	40	60

**Figure 1:**
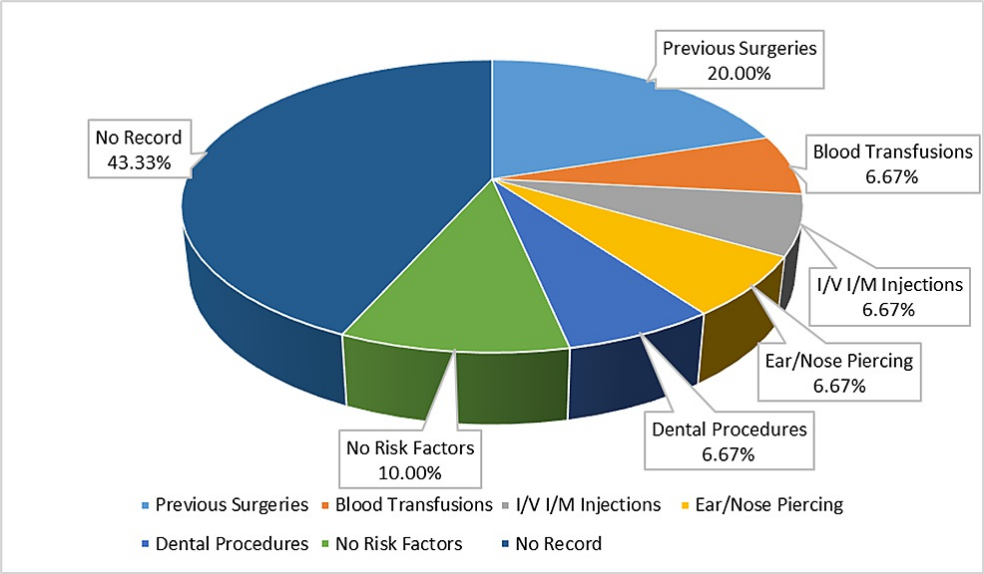
Risk factors.

Of 30, six patients (20%) had a history of previous surgeries, two (6.6%) had blood transfusions, two (6.6%) had dental procedures, two (6.6%) had IV or intramuscular (IM) injections, two (6.6%) had ear/nose piercing, and three (10%) had no risk factors. A total of 13 patients (43.3%) were unable to report a history of any of these risk factors (Figure 1).

Of 30, 16 patients (26.6%) had mild symptoms of anorexia, nausea, abdominal pain, constipation, fatigue, and body aches. The rest of the patients (73.4%) had no symptoms. In eight patients, pre-treatment liver function tests (LFTs), especially alanine aminotransferase (ALT) and aspartate aminotransferase (AST), were in the normal range (0-55 and 5-34), respectively. Four children received peginterferon and ribavirin out of these eight patients with normal LFTs. While in 22 patients, pre-treatment aminotransferases levels were above the normal range. They ranged from (ALT: 55-90 U/L), (AST: 5-34 U/L), (ALT up to 90 U/L), (AST up to 60 U/L), and (ALT and AST: >100U/L), in nine, eight, and five patients, respectively. However, they steadily declined to normal by the end of treatment.

Table 2 presented results from two reliable normality tests, namely the Kolmogorov-Smirnov test and the Shapiro-Wilk test. We used Shapiro-Wilk test due to the relatively small sample size. The table showed clearly that the dependent variables "pre and post-treatment ALT & AST" had non-normal distribution as the sig-value is below 0.05. We also applied the non-parametric Wilcoxon Signed Rank Test, which revealed that the p-values of both variables were also less than 0.05.

**Table 2:** Normal distribution of effect of treatment (sofosbuvir and ribavirin) on liver enzymes. ALT: Alanine aminotransferase; ALT: aspartate aminotransferase.

Variables	P-value
Pre and post-treatment ALT	0.000
Pre and post-treatment AST	0.000

Abdominal ultrasound of only 15 symptomatic patients was done, out of which only one had mild coarse liver, and the rest had normal findings. The status of liver fibrosis was assessed in patients by shear wave ultrasound. Of those assessed, two patients had an F=01 stage of hepatic fibrosis, one patient had an F=02 stage of hepatic fibrosis, and one patient had an F=03 stage of hepatic fibrosis. The rest had normal findings (Table 3).

**Table 3:** Findings on imaging.

	Ultrasound			Shear wave elastography			
	Normal	Mild coarse liver	Not done	Stage 1 fibrosis	Stage 2 fibrosis	Stage 3 fibrosis	Not done
Number	18	7	5	2	1	1	26
Percentage	60	23.3	16.7	6.66	3.3	3.3	86.6

**Figure 2:**
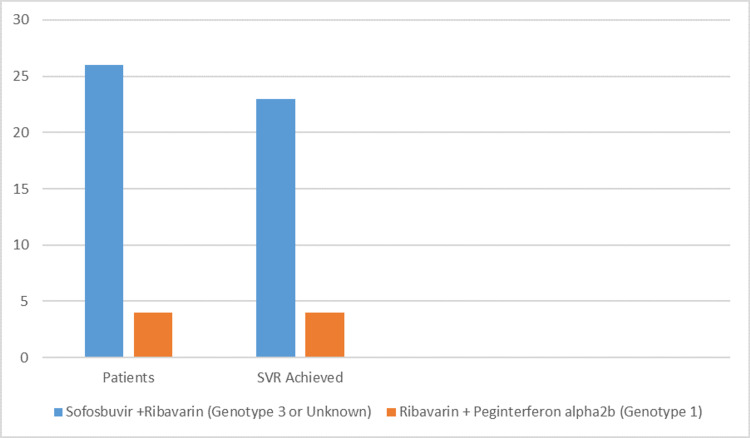
SVR achieved at six months of treatment. SVR: Sustained viral response.

Of these 30 patients, four had genotype 1, and 26 had genotype 3. The children with genotype 1 were treated with ribavirin and Peg-IFN-α-2b, and the remaining 26 children with genotype 3 were treated with sofosbuvir and ribavirin for 24 weeks. The dose was adjusted according to the weight. No significant adverse effects were noted during treatment. Post-treatment LFTs were normal in almost all patients. Quantitative HCV PCR was performed at 4, 12, and 24 weeks after initiating treatment. Sustained viral response (SVR) was achieved at six months in 27/30 (90%) patients. A total of 100% in genotype 1 (a+b) and 88.4% in genotype 3 patients achieved SVR (Figure 2).

## Discussion

HCV genotype 3 was most prevalent, followed by HCV genotype 1, in children included in our study. A systemized literature search carried out in Pakistan during 2009-2018 similarly reported that the prevalence of HCV genotype 3 was highest in the country, followed by untypable genotype and genotype 1 [6].

The outcomes of our study showed that the combination of sofosbuvir and ribavirin effectively treats HCV genotype 3 infection in children, if given in adequate doses and compliance ensured. All 23 patients with genotype 3 successfully achieved SVR at 24 weeks, except three patients who lost follow-up due to unaffordability and financial constraints. Overall, 90% of children with genotype 3 treated with this regime for 24 weeks achieved SVR. These results were comparable to those found by Wirth S et al. with SVR achieved of 97% [7]. Rosenthal P et al. also postulated that the sofosbuvir and ribavirin combination is safe and highly efficacious for HCV genotype 3 and 4 and reported the SVR achieved of 98% [8]. Furthermore, the result of our study was comparable to the findings of another research study carried out in Pakistan by Hashmi M and Cheema H, where the SVR achieved was 97% [9].

On the contrary, Peg-IFN-α-2b and ribavirin combination was also effective in treating HCV genotype 1 infection in children under 15 years. This combination remained the standard for treatment of HCV genotype 1 infection in children under 12 years of age for almost a decade. It was the only approved regime by EMA and FDA in this age group till September 2019 [10,11,12,13]. All four children in our study with HCV genotype 1 treated with peginterferon alfa 2a and ribavirin had undetectable HCV RNA by 24 weeks of treatment. Jara P et al. and Sokal EM et al. reported SVR of 44% and 57%, respectively, while Wirth S et al. stated the SVR of 53% in children with HCV genotype 1, treated with this combination [13,14,15]. SVR achievement in our study was far superior that in these studies, could be attributable to the small sample size of only four patients included in our study and mandates the need for more studies with a better sample size for elaboration.

Although all subjects included were otherwise healthy and did not have any significant signs or symptoms, the treatment improved their overall health, growth, and wellbeing. In addition, all patients tolerated the treatment well, and none of them was excluded due to drug intolerance or their side effects.

Most of the children had elevated aminotransferase levels prior to commencement of treatment which steadily declined to normal by the end of treatment. While most of the patients had unremarkable findings on abdominal ultrasound and stage F0 of hepatic fibrosis on shear wave elastography, four out of 30 patients had evidence of fibrosis on shear wave elastography ranging from stage F1 to F3 at presentation, which resolved completely at the completion of treatment. The resolution of fibrosis with HCV treatment on shear wave elastography has been reported earlier, hence reinforced by our study [16].

We also inferenced that majority of our patients contracted HCV infection from the use of secondhand syringes, unsterilized surgical instruments and dental procedures, poorly screened blood transfusions, and unhygienic settings of barbershops. HCV genotype 3 was found to be the most prevalent HCV genotype in Punjabi children, followed by HCV genotype 1. These results were similar to those found in other literature available [6,17].

With the everyday increasing armamentarium in HCV treatment and especially the recent approval for direct-acting antivirals (DAAs) in children aged 3-12 years by FDA, American Association for the Study of Liver Diseases (AASLD), and Infectious Diseases Society of America (IDSA), a new horizon for HCV treatment in the pediatric population is set [10,13]. However, besides a small sample size, one other limitation of our study was that it was carried out in an era just before the recommendation of these orally given and well-tolerated regimes for HCV treatment in children under 12 years.

Although the regime of peginterferon alfa and ribavirin showed excellent results in our study, with the new recommendations of interferon and ribavirin free regimes with pan-genotypic efficacy and shorter duration of treatment with high safety profiles, there is little room left for these conventional therapies in the coming future and the direction of treatment is needed to be tailored accordingly in a dynamic way [10,11,13].

## Conclusions

Hepatitis C is generally benign in children. However, early recognition and appropriate treatment are integral in preventing long-term complications in adults and taking positive steps towards hepatitis C eradication. Despite overall impressive results obtained in our study, we also acknowledge that with continuous drug development and increasing availability of DAAs for younger children, the landscape of HCV treatment is completely changed. Therefore, newer studies to evaluate immediate and long-term effects of these agents in children are intently desired.
